# Digitale Gesundheits-Apps: Wie gelingt die Integration in die medizinische Versorgung

**DOI:** 10.1007/s00108-024-01774-4

**Published:** 2024-10-10

**Authors:** Alexandra Widmer

**Affiliations:** Docs Digital, Hagenbeckstr. 37, 22527 Hamburg, Deutschland

**Keywords:** Digitalisierung, Künstliche Intelligenz, Arzt-Patienten-Kommunikation, Implementierung von Apps, Gesundheitsmonitoring, Digitalization, Artificial intelligence, Doctor–patient communication, Mobile applications, Health monitoring

## Abstract

Digitale Gesundheits-Apps, seit 2019 auch auf Rezept erhältlich, bieten vielfältige Funktionen in Gesundheitsmonitoring, Symptomerkennung, Verlaufsbeobachtung und Patientenversorgung. Das Digitale-Versorgungs-Gesetz von 2023 strebt eine umfassende Integration von Apps in die medizinische Versorgung an. Die Nutzung künstlicher Intelligenz (KI) in Gesundheits-Apps verspricht eine frühere Diagnosestellung, sensitive Symptomüberwachung, Optimierung von Therapieabläufen, effektivere Arzt-Patienten-Kommunikation sowie Ressourceneinsparungen. Die Integration von Gesundheits-Apps und KI kann eine stärker personalisierte, hochwertige Versorgung ermöglichen.

## Abstract (en)

Digital health applications (apps) have been available on prescription since 2019 and offer a multitude of functions in health monitoring, symptom recognition, follow-up monitoring and patient care. The Digital Care Act from 2023 strives for a comprehensive integration of apps into medical care. The utilization of artificial intelligence (AI) in health apps promises an earlier diagnosis, sensitive symptom monitoring, optimization of courses of treatment, more effective doctor-patient communication and saving of resources. The integration of health apps and AI can enable a stronger personalized high-value care.

Seit Inkrafttreten des Digitale-Versorgung-Gesetzes (DVG) 2019 haben gesetzlich Versicherte Anspruch auf eine kostenfreie Versorgung mit bestimmten „Gesundheits-Apps auf Rezept“, die von Ärzten und Psychotherapeuten verordnet werden können. Das Verzeichnis des Bundesinstituts für Arzneimittel und Medizinprodukte (BfArM; [1]) listet 56 digitale Gesundheitsanwendungen (DiGA). Hiervon unabhängig ist eine Vielfalt gesundheitsbezogener Anwendungen im Netz verfügbar. Übergreifende oder erkrankungsspezifische Gesundheits-Apps auf Mobilgeräten tragen Gesundheitsmonitoring, Warnfunktionen, Symptomerkennung, Risikoeinschätzung und Verhaltensanleitungen in den Alltag.

Am 14.12.2023 hat der Bundestag das „Gesetz zur Beschleunigung der Digitalisierung des Gesundheitswesens“ (DigiG) beschlossen. Ziel der zugrunde liegenden Initiative ist es, mit digitalen Lösungen die medizinische Versorgungsqualität zu verbessern. Nun soll es also auch in Deutschland möglich werden, die vollständige Patientenakte systematisch mit peripheren Datenquellen, Erhebungsinstrumenten und Monitoringsystemen zu vernetzen. Das zentrale Element dieser integrierten digitalen Gesundheitsversorgung ist die für 2025 für alle gesetzlich Versicherten avisierte elektronische Patientenakte (ePA; [2]). Hier sollen alle Informationen und Daten eines Menschen zusammenlaufen. Doch bereits heute ist eine Implementierung zur Verfügung stehender Informationen aus sogenannten „Gesundheits-Apps“, „Wearables“ und Ähnlichem in der medizinischen Versorgung möglich.

## Vielfältige Gesundheits-Apps bereits verfügbar

Gesundheits-Apps bieten beispielsweise die Möglichkeit der eigenständigen Überwachung von spezifischen Aspekten des Gesundheitszustands. Zudem können sie zunehmend in den Informations- bzw. Datenaustausch zwischen Ärzten und Patienten integriert werden.

Die wachsende Nutzung von Gesundheits-Apps bietet also ein hohes Potenzial für eine effektivere und effizientere Gesundheitsversorgung. Dies kann die persönliche Autonomie der Menschen stärken und lässt eine intensivierte Kooperation zwischen medizinischem Fachpersonal und Patienten erwarten.

Die AWMF befürwortet die Berücksichtigung der Versorgungsleitlinien in Gesundheits-Apps

Die Arbeitsgemeinschaft der Wissenschaftlichen Medizinischen Fachgesellschaften (AWMF) befürwortet zwar die Berücksichtigung der Versorgungsleitlinien in den eingesetzten Gesundheits-Apps, hält sich allerdings in ihren Leitlinien bei den Empfehlungsgraden für die Anwendung von Gesundheits-Apps noch zurück. Deren Einsatz ist in der Regel mit „Kann“-Empfehlungen spezifiziert [3]. Qualitätsprinzipien der AWMF für Gesundheits-Apps umfassen

- Transparenz,
- Zweckmäßigkeit,
- Angemessenheit der Risiken,
- ethische Unbedenklichkeit,
- Rechtskonformität,
- Validität und Aktualität der Informationen,
- technische Angemessenheit sowie
- Gebrauchstauglichkeit und Ressourceneffizienz [4].

Die zu erwartende Einbindung von künstlicher Intelligenz (KI) in Gesundheits-Apps verspricht dabei eine wesentliche Steigerung der Sensitivität und Spezifität sowie ein hohes Niveau an alltagsgerechten und intuitiv nutzbaren Interaktionsmöglichkeiten [5].

## Beitrag künstlicher Intelligenz

KI kann im Rahmen von Gesundheits-Apps zahlreiche Beiträge leisten, etwa um

- Diagnosen früher, schneller und genauer zu stellen,
- Risikofaktoren zu identifizieren,
- den Informationsaustausch zwischen Arzt und Patient zu verbessern,
- Prozesse wie Terminplanung und Dokumentation zu vereinfachen und zu optimieren,
- Muster oder Zusammenhänge zu erkennen und somit Diagnostik, Therapie und Forschung zu unterstützen,
- zur medizinischen Entscheidungsfindung beizutragen, etwa indem Leitlinien berücksichtigt werden,
- die Beziehung zwischen Arzt und Patient zu unterstützen, etwa mit personalisierten Empfehlungen,
- Zeit und Ressourcen zu sparen.

## Frühes Erkennen potenzieller Gesundheitsprobleme

Voraussichtlich wird die Anwendung von KI im medizinischen Bereich zunehmend gesetzlich reguliert, um die Transparenz der Funktion und die Sicherheit der Anwendung sicherzustellen.

Der Nutzen von Gesundheits-Apps hängt entscheidend von der regelmäßigen und korrekten Nutzung ab

Der Nutzen von Gesundheits-Apps hängt zudem entscheidend von der regelmäßigen und korrekten Nutzung sowie einer sorgfältigen Integration in die Gesundheitsversorgung der einzelnen Menschen ab. Eine zentrale Rolle für die Akzeptanz bei Ärzten spielt die Überzeugung, dass sich der Einsatz von Gesundheits-Apps positiv auf Gesundheit und Wohlbefinden der Patienten auswirken wird. Generell wünschen sie sich mehr Informationen und Schulungsmaterial [6].

Unstrittig ist, dass ein kontinuierliches Monitoring von Gesundheitsdaten und des Verhaltens mittels Gesundheits-Apps die frühere Erkennung von Auffälligkeiten oder potenziellen gesundheitlichen Problemen ermöglichen kann (Infobox 1). Dies kann zu rechtzeitigen Interventionen oder Verhaltensänderungen führen, die dann beispielsweise schwerwiegendere Erkrankungen und Verläufe verhindern.

Als niederschwelliger Einstieg in die Sphäre der Gesundheits-Apps bieten sich kostenfreie, breit verfügbare Anwendungen zur Überprüfung und Einordnung von Symptomen bei akuten oder chronischen Erkrankungen an oder Anwendungen zur Identifikation eines erhöhten Risikos für schwere Verläufe bei Infektionskrankheiten und zur Nennung leitlinienbasierter Optionen zum weiteren Prozedere, etwa bei „coronavirus disease 2019“ (COVID-19; [7]).

Auch über die Besprechung der von Smartwatches bereits ohne dedizierte Gesundheits-Apps erhobenen „Fitness“-Daten in der Sprechstunde können Hausärzte ihren Patienten den Einstieg in die Sphäre von Gesundheits-Apps bahnen.

### Infobox 1 Digitale Apps für Selbstdiagnostik und -monitoring

Symptomorientierte Anwendungen bieten Nutzern die Möglichkeit, ihre Beschwerden einzugeben. Sie erhalten dann Angaben zu möglichen Ursachen sowie Empfehlungen für potenzielle Verhaltensänderung und gegebenenfalls zur Notwendigkeit, einen Arzt aufzusuchen oder unmittelbar zu kontaktieren. Für digitale Symptombewertungs-Tools, die auf einzelne Erkrankungen ausgerichtet sind, wurde eine hohe Übereinstimmung mit der ärztlichen Beschreibung von Symptomen berichtet [8].

*Monitoring*: Wearables wie Smartwatches können vitalphysiologische Daten kontinuierlich monitoren, gegebenenfalls Alarme auslösen und/oder Ärzten in Echtzeit Feedback geben. Dies ermöglicht eine kontinuierliche Überwachung einzelner potenziell kritischer Parameter ohne hochfrequente physische Arztbesuche. Die Daten könnten Ärzten dabei helfen,

- zeitnah pathologische Auffälligkeiten festzustellen, weiterführende Diagnostik zu veranlassen und Behandlungsentscheidungen zu treffen oder
- individuelle Empfehlungen für einen gesünderen bzw. an eine chronische Erkrankung adaptierten Lebensstil abzugeben.

Beispielsweise bei Blutzucker- und Blutdruckmessungen könnten Messergebnisse automatisch in Echtzeit an die elektronische Patientenakte bzw. an den Arzt gesendet werden.

*Fitness- und Ernährungs-Apps*: Plattformen unterstützen Patienten dabei, ihre körperliche Aktivität und Ernährungsgewohnheiten zu monitoren. Hierzu gehören beispielsweise auch Apps zur Umsetzung regelmäßiger körperlicher Aktivität oder personalisierter Ernährung.

*Risikoscreening*: Spezifisch auf einzelne Erkrankungen bezogene Apps können das Risiko des Entstehens oder des Fortschreitens einer Erkrankung oder die Notwendigkeit einer Arztkonsultation zur weiterführenden Abklärung anzeigen.

*Management von Symptomen*: Syndrom- oder symptomorientierte Gesundheits-Apps können beispielsweise beim Umgang mit chronischen Erkrankungen oder Zuständen, etwa beim menopausalen Syndrom, positive Effekte haben [9].

*Identifikation von Nebenwirkungen und Wechselwirkungen*: Basierend auf entsprechenden Symptomen lassen sich Nebenwirkungen von Therapien auch im Alltag monitoren. Auch Warnhinweise für ungeeignete Medikamentenkombinationen sind eine wichtige Anwendung.

## Implementierung in die Arzt-Patienten-Kommunikation

Digitale Lösungen und KI-gestützte Systeme könnten die medizinische Ausbildung und Praxis ebenso grundlegend verändern wie die Kommunikation zwischen Ärzten und Patienten (Infobox 2). Dabei ermöglicht die Integration von Gesundheits-Apps neue Wege einer stärker personalisierten, qualitativ hochwertigen und patientenzentrierten Gesundheitsversorgung. Hierzu müssen alle genutzten Anwendungen unter Beachtung der geltenden medizinischen und informationellen Standards implementiert werden, um die Anwendungssicherheit und den persönlichen Datenschutz zu gewährleisten.

Derzeit bestehen noch gewisse Hürden bei der Integration von Gesundheits-Apps und deren Daten in die Routineversorgung. Dabei spielen unter anderem die Schnittstellen zu Patientenmanagementsystemen und Fragen der Vergütung eine Rolle. Aufseiten der Patienten können vor allem bei Älteren Unkenntnis und Vorbehalte als Hemmnisse erscheinen, die sich jedoch in der Regel überwinden lassen. Digitale Fähigkeiten können sich unabhängig vom Alter entwickeln.

Die Grundlage für eine entsprechende Beratung ist die „Digitalanamnese“, etwa anhand der folgenden Fragen:

- Welche digitalen Geräte nutzen Sie regelmäßig – Handy, Tablet, Computer?
- Wofür verwenden Sie diese Geräte hauptsächlich – Telefonate, Messaging, Videos, Chatten, Lesen, Arbeiten?
- Wo suchen Sie normalerweise nach Gesundheitsinformationen – allgemein im Internet, auf bestimmten Plattformen, mittels KI-gestützter Chatbots, auf offiziellen bzw. validierten Gesundheitsseiten?
- Nutzen Sie bereits Gesundheits-Apps aus einem App-Store? Wurden Ihnen solche Apps von Ihrer Krankenkasse empfohlen?
- Besitzen Sie eine Smartwatch? Wenn ja, wofür nutzen Sie diese?
- Haben Sie bereits irgendwelche Ihrer Gesundheitsdaten digital verfolgt oder erfasst – etwa Schrittzahl, Blutzuckerspiegel, Schlafmuster, Blutdruck, Stimmungslage?

Die so erhobenen Informationen können dazu beitragen, die individuell am besten geeigneten digitalen Lösungen zu finden, die bei Nutzung zwischen den Arztbesuchen die Versorgung verbessern können – und das bereits jetzt und heute.

Die Qualität der Patientenversorgung kann erheblich profitieren, wenn Patienten mit Gesundheits-Apps aktiv ihre eigene Gesundheit monitoren und ihr Verhalten steuern – und wenn Ärzte als verlässliche Begleiter im Zeitalter der digitalen Medizin agieren.

### Infobox 2 Möglichkeiten zur Implementierung von Gesundheits-Apps in die Arzt-Patienten-Kommunikation

Digitale Gesundheits-Apps tragen strukturierte Gesundheitsversorgung in den Alltag. Durch die Integration von deren Daten – die auch in die elektronische Patientenakte aufgenommen werden könnten – sind Ärzte in der Lage, Informationen über den aktuellen Gesundheitszustand ihrer Patienten in Echtzeit zu prüfen bzw. jederzeit darauf zuzugreifen. Dies kann fundiertere Entscheidungsprozesse ermöglichen und die effektive Beratung während der zeitlich meist eng limitierten Praxistermine unterstützen [10].

Folgende Anwendungen stehen im Vordergrund:

- *Risikoidentifikation*: Von Gesundheits-Apps unterstützte Patientenprofile können zur niederschwelligen Identifikation individueller Erkrankungs- und Therapierisiken dienen.
- *Telemedizinische Konsultationen*: Gesundheits-Apps können als Grundlage für telemedizinische Konsultationen dienen. Ärzte können dabei auch die von den Patienten erfassten Daten analysieren und in der Folge über virtuelle Plattformen Ratschläge erteilen oder Anpassungen des Behandlungsplans vornehmen.
- *Behandlungsoptionen*: Mit Gesundheits-Apps, die über verschiedene verfügbare Therapiemöglichkeiten informieren, können Patienten mit geeignetem Vorwissen in das Arztgespräch gehen, um gemeinsam fundiertere Entscheidungen zu treffen.
- *Patientenschulung*: Gesundheits-Apps können als Schulungsinstrumente dienen, um Patienten über ihren Gesundheitszustand, sinnvolle Verhaltensweisen und den zweckmäßigen Gebrauch von Medikamenten oder Geräten zu unterrichten. Ärzte würden dann für die längerfristige digitale Unterstützung Apps empfehlen, die ihren Patienten beim Management chronischer Erkrankungen helfen können.
- *Feedback und Follow-up*: Basierend auf der regelmäßigen Kontrolle von Daten der Gesundheits-Apps können Ärzte automatisiertes Feedback oder gegebenenfalls personalisierte Rückmeldungen geben und beispielsweise den Behandlungs- oder Maßnahmenplan bedarfsgerecht anpassen. Dies fördert eine kontinuierliche Interaktion von Arzt und Patient, auch zwischen den Arztbesuchen.
- *Medikationsmanagement*: Gesundheits-Apps helfen Patienten, ihre eigene Medikamenteneinnahme zu monitoren. Die Apps können Erinnerungssignale setzen und Benachrichtigungen auslösen, wenn es zu Problemen mit der Adhärenz kommt.
- *Management chronischer Erkrankungen*: Am Beispiel Diabetes mellitus erklärt unterstützen Gesundheits-Apps nicht nur die Blutzuckerüberwachung, sondern können auch Mahlzeiten und Insulinzufuhr aufzeichnen. Ärzte können die Daten analysieren und individuelle Anpassungen des Diabetesmanagementplans vornehmen und gegebenenfalls Verhaltensänderungen vorschlagen.
- *Förderung gesundheitlich sinnvollen Verhaltens*: Gesundheits-Apps können unter anderem durch Bewusstmachung des eigenen Verhaltens einen aktiven und gesundheitlich förderlichen Lebensstil unterstützen. Dabei ist ein integrierter Ansatz mit Techniken zur Verhaltensveränderung von entscheidender Bedeutung. Hierzu zählen konkrete Zielsetzungen, Selbstmonitoring, Feedback und sozialer Support, der durch gemeinsame Nutzung in Gruppen oder Communities begünstigt wird [11].
- *Integration von Wearables*: Wearables wie Fitness-Tracker und Smartwatches können in die Anwendung von Gesundheits-Apps integriert werden. Die Geräte liefern kontinuierliche Daten zu Verhalten und Lebensgewohnheiten der Patienten, die der Arzt in die Gesamteinschätzung einbeziehen kann.

## Fallvignette

Eine Patientin kommt aufgeregt in die Notfallambulanz und berichtet, ihre Gesundheits-App zeige seit einer Woche immer wieder an, sie solle dringend mit ihrem Arzt sprechen, da sie in der letzten Zeit zu wenig Schlaf bekomme. Obwohl sie sich subjektiv wohlfühlt, ist sie nun der Aufforderung gefolgt. Tatsächlich ist anhand der Aufzeichnungen der Smartwatch erkennbar, dass sie in den letzten sieben Nächten sehr wenig geschlafen hat – jeweils höchstens zwei Stunden.

Die Patientin steht wegen einer manisch-depressiven Erkrankung schon längere Zeit unter medikamentöser Behandlung mit Phasenprophylaktika. In der Vergangenheit führten manische Episoden oft zu Klinikaufenthalten, monatelanger Arbeitsunfähigkeit und längerer Betreuung ihrer Kinder in Pflegefamilien. Mit der sofortigen Umstellung der Medikation kann eine sich ankündigende manischen Phase kupiert werden.

## Herausforderungen der breiten Implementierung

Viele Ärzte stehen DiGA aktuell noch skeptisch gegenüber, meist aufgrund mangelnder Erfahrung mit digitalen Tools, aber auch wegen Vorbehalten hinsichtlich des medizinischen Nutzens der Apps. Hier gilt es, Ärztegremien früher und stärker in die Entwicklung einzubeziehen.

Wichtige Hürden für die Akzeptanz von Gesundheits-Apps in der Versorgungspraxis sind zunächst technische Hemmnisse, insbesondere die Kompatibilität von Hardware, Software und Betriebssystemen sowie der Bedarf an standardisierten Schnittstellen zur Integration von DiGA und ihrer generierten Daten in bestehende Praxismanagementsysteme und ePA.

Aktuell fehlen noch breit konsentierte Kriterien zur Bewertung von Wirksamkeit und Sicherheit

Die Beurteilung der Qualität einer Gesundheits-App muss zahlreiche Aspekte berücksichtigen. Aktuell fehlen noch breit konsentierte Kriterien zur Bewertung von Wirksamkeit und Sicherheit, sodass die Qualitätsstandards variieren.

In Deutschland regelt das DVG die Zulassung und Erstattung von DiGA durch Krankenkassen. Anwendungen höherer Risikoklassen (II und III) unterliegen einer intensiveren Prüfung durch das BfArM, um die Sicherheit und den klinischen Wert sicherzustellen. Die Prüfung umfasst folgende Aspekte:

- Nachweis von Sicherheit und Nutzen anhand klinischer Daten gemäß Medical Device Regulation
- CE-Kennzeichnung, umfassende Dokumentation sowie Prüfung durch eine behördlich beauftragte Organisation
- Einhaltung der Datenschutz-Grundverordnung (DSGVO)
- Integration mit Gesundheits-IT-Systemen
- Barrierefreiheit und Benutzerfreundlichkeit

Trotz strenger Vorschriften der DSGVO bestehen weiterhin Risiken durch Cyberangriffe oder unsachgemäße Handhabung von Daten durch Anbieter oder Anwender. Es bedarf also der koordinierten Zusammenarbeit von Entwicklern, Behörden, medizinischen Fachkräften und Nutzern, um das Potenzial von DiGA für die Patientenversorgung optimal zu erschließen.

## Studien zur erfolgreichen Integration digitaler Gesundheitsanwendungen in den Versorgungsalltag im Rahmen der Arzt-Patienten-Kommunikation

Bisher ist die Studienlage zur Integration digitaler Lösungen in den medizinischen Versorgungsalltag auf weitestgehend spezifische Szenarien und Produkte beschränkt. Einen umfangreichen, wissenschaftlichen Überblick zu dieser Thematik lässt der aktuelle Forschungsstand nicht zu, weshalb im Folgenden beispielhaft Studien zur Anwendung von Apps in bestimmten Indikationen beschrieben werden.

Eine App-basierte pulmonale Rehabilitation bei chronisch-obstruktiven Lungenerkrankungen ist im Vergleich zu konventionellen Maßnahmen mit günstigen Ergebnissen in Bezug auf körperliche Leistungsfähigkeit, Symptomatik, Lebensqualität und Hospitalisierung assoziiert. Sie kann eine vorteilhafte Option sein, wenn eine konventionelle zentrumsbasierte Rehabilitation nicht durchführbar ist [12].

Apps zum Monitoring des Gangmusters bei Menschen mit multipler Sklerose zeigen eine hohe Korrelation mit dem Behinderungsgrad und könnten zur Detektion einer Erkrankungsprogression mit erforderlicher Therapieumstellung beitragen [13]. Apps zur Messung von Mobilität und Geschicklichkeit im Alltag ergänzen die realitätsnahe Überwachung des Funktionsstatus [14], ebenfalls mit dem Ziel eines rechtzeitigen Therapiewechsels bei Versagen der laufenden Therapie.

Die Smartphone-basierte „FiApp“ (Google, Mountain View, CA, USA) dient dazu, Patienten mit langfristiger Hämodialyse bei der Überwachung und Steuerung ihrer Flüssigkeitsaufnahme zu unterstützen. Motivierte Patienten sind damit in der Lage, die kardiovaskulär belastende Wassereinlagerung zwischen den Dialyseterminen in klinisch relevantem Umfang zu reduzieren [15].

## Ausblick

Die weitere Implementierung digitaler Lösungen in den medizinischen Alltag birgt großes Potenzial (i) zur Optimierung der patientenindividuellen Versorgung mit entsprechender Verbesserung der gesundheitlichen Situation und (ii) zur effektiveren Versorgung großer Bevölkerungsgruppen. Offenheit gegenüber neuen digitalen Möglichkeiten, sowohl vonseiten der Ärzte als auch vonseiten der Patienten, könnte dabei helfen, die medizinische Versorgung zukunftsweisend zu gestalten und kontinuierlich zu optimieren.

### Mehr Informationen zum Thema

https://www.diga-verzeichnis.de/

https://www.vfa.de/de/wirtschaft-politik/pharma-digital/diga-watchlist

https://gesund.bund.de/digitale-gesundheitsanwendungen-diga

## Fazit für die Praxis

- Der Bundestag hat Ende 2023 die beschleunigte Digitalisierung des Gesundheitswesens gesetzlich verankert.
- Gesundheits-Apps können bereits heute vielseitig in die medizinische Versorgung einbezogen werden. Die Arbeitsgemeinschaft der Wissenschaftlichen Medizinischen Fachgesellschaften (AWMF) hat hierfür Qualitätsprinzipien definiert.
- Die zusätzliche Integration von künstlicher Intelligenz in die Arzt-Patienten-Kommunikation eröffnet das Potenzial für eine stärker personalisierte und patientenzentrierte Gesundheitsversorgung, frühere Diagnosen und mehr Autonomie.
- Unterstützt durch positive Ergebnisse aus Studien zur Nutzung von Gesundheits-Apps in medizinischen Szenarien kann die Öffnung für die rasch expandierenden digitalen Möglichkeiten die Versorgungslandschaft zukunftsweisend optimieren.
